# Quinine Found in the Urine and in the Blood

**Published:** 1842-11-05

**Authors:** 


*Quinine found in the Urine and in the blood.*—M. Landerer, on examining the sediment from the urine of a patient, to whom the sulphate of quinia had been given for intermittent neuralgic pains, found, besides the phosphate and urate of lime and carbonate of ammonia, traces of free quinia; the urine contained quinia in addition to the carbonate, sulphate, and hydrochlorate of ammonia.

The blood of a patient who had been taking quinia for remittent fever, and which was drawn on account of the supervention of pleurisy, had but a slightly bitter taste when first drawn, and while still warm; the bitterness was much more marked when it had become cold and coagulated. There was a great difference in the taste of the serum and of the coagulum; the latter was scarcely bitter, while the former was very much so. The serum consequently yielded quinia, after having been evaporated, the residue digested with acidulated water, filtered, and precipitated by ammonia. The fæces also contained quina. The serum of the blood of another patient also yielded quinia on analysis, presenting results exactly similar to those afforded by the first patient.—*Ibid, from Repertorium für Pharmacie.*

